# Challenges of self-reported medical conditions and electronic medical records among members of a large military cohort

**DOI:** 10.1186/1471-2288-8-37

**Published:** 2008-06-05

**Authors:** Besa Smith, Laura K Chu, Tyler C Smith, Paul J Amoroso, Edward J Boyko, Tomoko I Hooper, Gary D Gackstetter, Margaret AK Ryan, the Millennium Cohort Study Team

**Affiliations:** 1 Department of Defense Center for Deployment Health Research at the Naval Health Research Center, USA; 2 Madigan Army Medical Center, Tacoma, WA, USA; 3 Seattle Epidemiologic Research and Information Center, Veterans Affairs Puget Sound Health Care System, Seattle, WA, USA; 4 Department of Preventive Medicine and Biometrics, Uniformed Services University of the Health Sciences, Bethesda, MD, USA; 5 Analytic Services, Inc. (ANSER), Arlington, VA, USA

## Abstract

**Background:**

Self-reported medical history data are frequently used in epidemiological studies. Self-reported diagnoses may differ from medical record diagnoses due to poor patient-clinician communication, self-diagnosis in the absence of a satisfactory explanation for symptoms, or the "health literacy" of the patient.

**Methods:**

The US Department of Defense military health system offers a unique opportunity to evaluate electronic medical records with near complete ascertainment while on active duty. This study compared 38 self-reported medical conditions to electronic medical record data in a large population-based US military cohort. The objective of this study was to better understand challenges and strengths in self-reporting of medical conditions.

**Results:**

Using positive and negative agreement statistics for less-prevalent conditions, near-perfect negative agreement and moderate positive agreement were found for the 38 diagnoses.

**Conclusion:**

This report highlights the challenges of using self-reported medical data and electronic medical records data, but illustrates that agreement between the two data sources increases with increased surveillance period of medical records. Self-reported medical data may be sufficient for ruling out history of a particular condition whereas prevalence studies may be best served by using an objective measure of medical conditions found in electronic healthcare records. Defining medical conditions from multiple sources in large, long-term prospective cohorts will reinforce the value of the study, particularly during the initial years when prevalence for many conditions may still be low.

## Background

Epidemiological studies often rely on self-reported medical history for both exposure and outcome information. A number of studies have addressed the reliability of these data by comparing self-reported information with objective sources, such as medical records. Results vary by study population and by diagnosis, as well as study design [1-13]. Previous research on the accuracy of self-reported angina shows low agreement (kappa [κ] = 0.57) in elderly patients [2], but substantial agreement (κ = 0.72) in men participating in the British Regional Heart Study [7], with differences possibly attributed to dissimilarities in study populations and/or study design [5,10,14]. Variability by medical condition within the same study population has also been noted [1,3,5,10,12]. In a study of chronic diseases in elderly patients, researchers found high rates of agreement between self-reported and recorded diagnoses using kappa statistics for diabetes (κ = 0.84) and hypertension (κ = 0.70), but moderate to poor agreement for chronic lung disease (κ = 0.55), osteoarthritis of the knee (κ = 0.54), and chronic low back pain (κ = 0.36) [12]. Several studies suggest that this variability may be due to poor communication between the health care provider and the patient, since diseases with clear diagnostic criteria (e.g., diabetes, hypertension, myocardial infarction) tend to have higher rates of agreement than those that may be more complicated to diagnose by the physician or more difficult for the patient to understand (e.g., heart failure) [1,3,10].

Using data from the Millennium Cohort Study, a longitudinal study designed to assess the long-term health effects of military service [15], self-reported clinician-diagnosed medical conditions were compared with diagnostic codes from available electronic medical records. Unlike previous studies of this kind, the current study investigated a constellation of medical conditions that, to the best of our knowledge, have not been previously examined. Understanding the potential limitations of self-reported versus objective medical record data for a broad array of medical conditions will yield greater understanding of the results of future epidemiological studies based on comparable data sources for similar health outcomes.

## Methods

### Study population

The Millennium Cohort Study is a large, 21-year prospective study aimed at evaluating the potential effects of deployment and other military occupational exposures on long-term health outcomes using self-reported and electronic military health care data [15,16]. The invited participants were randomly selected from over 2 million US military personnel on active rosters in October 2000, with oversampling of Reserve and National Guard personnel, female service members, and those recently deployed, to ensure adequate statistical power to detect differences in even relatively rare outcomes in these subgroups. The baseline enrollment ended with 36% of those invited consenting to participate in the 21-year study. When compared with the 2000 US military at large, Cohort members were slightly more likely to be female, older, better educated, married, officers, in the Air Force, and from health care occupations [15]. The higher enrollment of women and those recently deployed reflects the intended oversampling [15]. Analyses to investigate potential reporting biases show no differential in responder health with respect to hospitalization and outpatient encounters in the year prior to enrollment [17], strong test-retest reliability [18], reliable vaccination reporting [19,20], occupation reporting [21], and deployment reporting [22] and minimal differences between participants choosing web submission in comparison to paper submission [23].

Demographic and military data for the Cohort, as of October 1, 2000, included sex, date of birth, education, marital status, race/ethnicity, previous deployment experience (January 1, 1998, to September 1, 2000), pay grade, service component (active duty and Reserve/Guard), service branch (Army, Navy/Coast Guard, Air Force, and Marine Corps), and occupation.

The population for this study consisted of participants from the first panel of Millennium Cohort participants who voluntarily consented and completed a baseline questionnaire (*n *= 77,047) between 2001 and 2003. Reservists and National Guard members (*n *= 39,028) were excluded because their electronic medical records are not fully available within the Department of Defense (DoD) medical record system. Additionally, Cohort members who failed to respond to any of the questionnaire items related to the medical conditions of interest (*n *= 124) or who had missing covariate data (*n *= 97) were excluded. The remaining 37,798 (49 percent of the first panel) comprise the study population for these analyses.

### Medical outcomes

The Millennium Cohort survey included a number of more serious diseases often associated with age [15]. Though the population was fairly young at baseline (54% of cohort members were younger than age 35), by the end of the 21-year study, many will have reached an age associated with increased risk for chronic diseases. Self-reported medical conditions listed in Additional file 1 were based on responses to the question: "Has your doctor or other health professional EVER told you that you have any of the following conditions?" "Yes" or "No" response choices were provided for each condition.

Individual, electronic hospitalization and ambulatory data included diagnoses using *International Classification of Diseases*, Ninth Revision, Clinical Modification (ICD-9-CM) codes [24]. These data were acquired from three sources: (1) the Standard Inpatient Data Record (SIDR), (2) the Standard Ambulatory Data Record (SADR), and (3) the Health Care Service Record (HCSR). SIDR contains up to eight ICD-9-CM discharge diagnoses for individual inpatient care at any DoD medical treatment facility worldwide since October 1988. SADR contains up to four ICD-9-CM diagnoses for individual outpatient encounters at any DoD health care facility since October 1998. HCSR contains up to 10 ICD-9-CM diagnoses for encounters at civilian facilities that are reimbursed by the DoD insurance system. These files contain historical inpatient data from October 1993 and outpatient data from October 1999. For each participant, all electronic data were scanned for ICD-9-CM codes corresponding to medical conditions from the time earliest records were available up to and including the date of survey submission.

In order to compare self-reported and electronic data, ICD-9-CM codes were selected to best represent the 38 medical conditions included in the questionnaire. Selection of one or more codes representing each medical condition evaluated was accomplished by several groups of paired clinician researchers, each blinded to the diagnostic codes selected by the other. Any discrepancies in ICD-9-CM codes selected were resolved through discussion. In addition, annual changes in ICD-9-CM coding up to 2003 (last year of survey submission) were accounted for in the final list of codes (Additional file 1) [25]. Electronic medical records were scanned in chronological order, and diagnostic fields were scanned in numerical order for the selected diagnostic codes. Any diagnostic code in any portion of the medical record indicated agreement with a self-reported medical condition.

### Statistical analysis

The prevalence of each condition was computed for both the self-reported and electronically maintained data. Statistical comparisons of these frequencies were performed using the chi-square test. Prevalence of conditions identified exclusively through the electronically maintained medical records was reported to estimate what might be lost by use of self-report alone.

Several measures of agreement were considered for interpretation of results in this study. An omnibus index, such as the kappa statistic, is often utilized in validity studies, but is appropriate only if the sole purpose of the research is to compare responses over time or to previous studies. If the results are intended for use in future studies, an omnibus index is unsatisfactory, and thus both measurements being compared should be presented. Furthermore, the kappa statistic is strongly affected by prevalence (i.e., when the prevalence is low, kappa approaches zero). Many of the medical conditions in the current study had low prevalence, therefore, the kappa statistic was not deemed appropriate for these analyses.

Sensitivity and specificity were considered as an alternative approach. Sensitivity and specificity might be used to explain how a positive or negative self-report of a particular medical condition compares with a documented diagnostic code in the medical record. However, these measures of diagnostic test performance become inappropriate to use when there is no "gold standard." Although the electronic medical records are thought to accurately reflect actual diagnoses for the active-duty study population, they are subject to coding errors and/or omissions, as well as potential biases, for example, those related to reimbursement issues. In addition, while participants are asked to consider their lifetime when answering the survey question, the electronic data only contain records beginning in October 1988. Furthermore, electronic data may not capture conditions diagnosed prior to active-duty military service. Since neither self-reported nor electronic medical record data were considered the gold standard for the existence of a medical condition, we chose not to report sensitivity and specificity.

After considering these alternatives, the present study used an approach similar to a previous investigation of cardiovascular patients in which positive and negative agreement was used to compare self-reported data on medical conditions with electronically available medical records [11]. Positive and negative agreement was selected as our analytic approach to resolve the omnibus issue [26] and the lack of a diagnostic gold standard. Positive and negative agreement, unlike the kappa statistic, is unaffected by imbalances in marginal totals caused by high or low prevalence. This approach may provide a better understanding for analyses based on various data sources, including insight into the limitations of both self-reported and objective electronic data pertaining to a large number of medical conditions.

**Figure 1:**
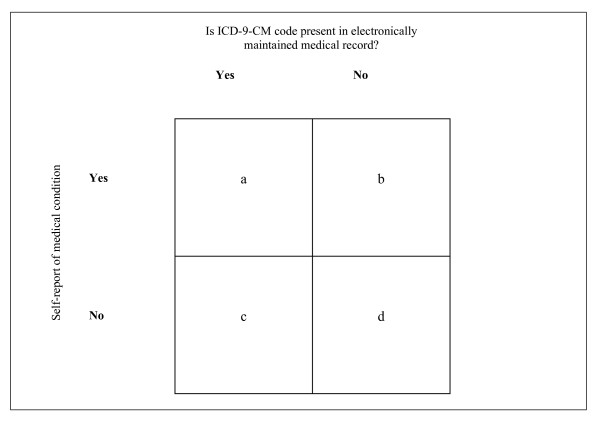
Illustration of the 2 × 2 table used to calculate positive and negative agreement.

Figure 1 illustrates the basis for calculating positive and negative agreement using a standard 2 × 2 table. Positive agreement was calculated as 2a/[N + (a - d)], where N = total observations [a + b + c + d]; and negative agreement was calculated as 2d/[N - (a - d)] [26]. The effect of length of service on agreement was also assessed, since individuals with longer time in service would be more likely to have diagnoses captured in military electronic medical records. Prevalence, as well as positive and negative agreement, was stratified by length of service using 5-year intervals (0–5, 6–10, 11–15, ≥ 16 years). All analyses were performed using SAS software (Version 9.1.3, SAS Institute, Inc., Cary, NC).

## Results

Self-reported medical conditions from questionnaire responses and objective health encounter data for a total of 37,798 Millennium Cohort participants were available for analysis. As previously described, participants who skipped all 38 medical conditions listed in the questionnaire (*n *= 124) were excluded from these analyses. Approximately 88 percent of the remaining answered all 38 conditions. In order to maximize the numbers available to assess each medical condition, participants failing to answer an individual medical condition were removed only from the analysis of that particular condition. This resulted in sample sizes that varied from 37,328 to 37,696 for each individual medical condition.

Of the 37,798 total participants, just over 50 percent reported ever being told by a health professional that they had at least one of the 38 medical conditions on the questionnaire. Statistically significant differences among those reporting at least one condition and those not reporting any condition were found for all demographic and military characteristics, except for military pay grade (data not shown). A higher proportion of women, black non-Hispanics, those who were married, of older age, and in health care and functional support occupations, self-reported at least one condition (Table 1).

**Table 1:** Demographic and military characteristics of active-duty Millennium Cohort participants (2001–2003) self-reporting medical conditions

Characteristic*	Study population^† ^*N *= 37,798 n (%)	Subjects who reported one or more conditions *n *= 18,581 n (%)
**Sex**		
Male	28,873 (76.4)	12,770 (68.7)
Female	8,925 (23.6)	5,811 (31.3)
**Age, years**		
17–24	8,334 (22.1)	3,274 (17.6)
25–34	14,324 (37.9)	6,263 (33.7)
35–44	13,048 (34.5)	7,578 (40.8)
>44	2,092 (5.5)	1,466 (7.9)
**Education**		
Some high school	1,072 (2.8)	540 (2.9)
High school graduate	18,845 (49.9)	9,062 (48.8)
Some college	10,470 (27.7)	5,159 (27.8)
College graduate	3,855 (10.2)	1,843 (9.9)
Advanced degree	3,556 (9.4)	1,977 (10.6)
**Marital status**		
Married	25,096 (66.4)	12,834 (69.1)
Not married	12,702 (33.6)	5,747 (30.9)
**Race/ethnicity**		
White non-Hispanic	24,926 (65.9)	12,306 (66.2)
Black non-Hispanic	6,153 (16.3)	3,178 (17.1)
Other	6,719 (17.8)	3,097 (16.7)
**Military pay grade**		
Enlisted	31,337 (82.9)	15,347 (82.6)
Officer	6,461 (17.1)	3,234 (17.4)
**Previous deployment experience**		
Nondeployed	21,788 (57.6)	11,265 (60.6)
Deployed	16,010 (41.4)	7,316 (39.4)
**Service branch**		
Army	14,355 (38.0)	7,110 (38.3)
Air Force	11,400 (30.1)	5,452 (29.3)
Navy/Coast Guard	9,331 (24.7)	4,824 (26.0)
Marines	2,712 (7.2)	1,195 (6.4)
**Length of service, years**		
0–5	7,231 (19.1)	2,909 (15.7)
6–10	8,571 (22.7)	3,554 (19.1)
11–15	10,250 (27.1)	4,939 (26.6)
≥ 16	11,746 (31.1)	7,179 (38.6)
**Occupational category**		
Combat specialists	7,505 (19.9)	3,277 (17.6)
Electronic equipment repair	3,926 (10.4)	1,927 (10.4)
Communications/intelligence	3,246 (8.6)	1,613 (8.7)
Health care specialists	3,279 (8.7)	2,021 (10.9)
Other technical & allied specialists	1,086 (2.9)	544 (2.9)
Functional support specialists	7,385 (19.5)	3,955 (21.3)
Electrical/mechanical	6,463 (17.1)	2,941 (15.8)
Craft workers	1,028 (2.7)	484 (2.6)
Service & supply handlers	2,956 (7.8)	1,470 (7.9)
Trainees, other	924 (2.4)	349 (1.9)

* Characteristic reflects status at time of study sample selection, October 2000.

^† ^Active-duty participants who answered at least one medical history question.

Of the 38 conditions, the most commonly noted from both data sources was sinusitis (Table 2). Other relatively common acute or transient medical conditions were migraine headaches and depression. Relatively common chronic medical conditions were hypertension and significant hearing loss. Prevalence based on self-report ranged from 0.5 percent for stroke and cirrhosis to 14.8 percent for sinusitis. A slightly lower range (0.2 percent to 13.9 percent, respectively) was found in the electronic medical records. Prevalence based on electronically recorded data was consistently lower than prevalence based on self-report for most conditions, with the exception of chronic bronchitis, manic-depressive disorder, schizophrenia or psychosis, and neuropathy-caused reduced sensation in the hands or feet. For medical conditions found exclusively in the electronic data, prevalence ranged from 0.0 percent for cirrhosis to 8.8 percent for sinusitis. Positive agreement values ranged from 1.0 percent for kidney failure to 58.2 percent for thyroid conditions. Negative agreement values were substantially higher, ranging from 89.2 percent for sinusitis to 99.7 percent for eight listed conditions, including heart attack, pancreatitis, and stroke.

**Table 2:** Prevalence, positive agreement, and negative agreement of active-duty Millennium Cohort participant self-reported and electronic medical record data

Medical condition*	% and 95% CI Self-reported	% and 95% CI Electronic- recorded	% Exclusively Electronic Recorded	Positive Agreement	Negative Agreement
**More Likely Chronic**					
Hypertension (high blood pressure)	10.4 (10.1, 10.7)	8.1 (7.8, 8.3)	3.1	53.5	95.3
Significant hearing loss	9.4 (9.1, 9.7)	5.9 (5.7, 6.1)	3.5	31.9	94.3
Chronic bronchitis	3.3 (3.1, 3.4)	4.0 (3.8, 4.2)	3.5	12.9	96.7
Sleep apnea	2.7 (2.6, 2.9)	1.5 (1.4, 1.6)	0.5	45.1	98.8
Rheumatoid arthritis	2.4 (2.2, 2.5)	0.3 (0.2, 0.3)	0.2	7.7	98.8
Thyroid condition other than cancer	2.2 (2.1, 2.4)	1.9 (1.8, 2.0)	0.7	58.2	99.1
Cancer	2.0 (1.9, 2.2)	1.8 (1.7, 2.0)	1.0	44.1	98.9
Chronic fatigue syndrome	1.3 (1.1, 1.4)	0.2 (0.1, 0.2)	0.1	5.1	99.3
Diabetes or sugar diabetes	1.2 (1.1, 1.3)	1.1 (0.9, 1.2)	0.6	37.4	99.3
Ulcerative colitis or proctitis	0.9 (0.8, 1.0)	0.3 (0.2, 0.3)	0.1	29.7	99.6
Manic-depressive disorder	0.9 (0.8, 0.9)	2.1 (2.0, 2.3)	1.9	11.8	98.7
Hepatitis C	0.8 (0.7, 0.9)	0.2 (0.2, 0.3)	0.1	25.4	99.6
Coronary heart disease	0.7 (0.6, 0.8)	0.6 (0.5, 0.6)	0.4	24.8	99.5
Emphysema	0.6 (0.5, 0.7)	0.2 (0.1, 0.2)	0.2	2.7	99.6
Lupus	0.6 (0.5, 0.7)	0.1 (0.1, 0.2)	0.1	17.4	99.7
Multiple sclerosis	0.6 (0.5, 0.7)	0.1 (0.1, 0.1)	0.0	14.1	99.7
Crohn's disease	0.6 (0.5, 0.7)	0.2 (0.2, 0.2)	0.1	17.1	99.7
Schizophrenia or psychosis	0.6 (0.5, 0.7)	0.8 (0.7, 0.8)	0.7	6.4	99.4
Kidney failure requiring dialysis	0.6 (0.5, 0.6)	0.0 (0.0, 0.0)	0.0	1.0	99.7
Cirrhosis	0.5 (0.5, 0.6)	0.0 (0.0, 0.0)	0.0	2.0	99.7
**More Likely Acute or Transient**					
Sinusitis	14.8 (14.5, 15.1)	13.9 (13.5, 14.2)	8.8	35.7	89.2
Migraine headaches	10.8 (10.5, 11.1)	4.3 (4.1, 4.5)	1.4	39.2	95.0
Depression	7.0 (6.8, 7.3)	6.7 (6.4, 6.9)	3.2	50.5	96.4
Bladder infection	6.9 (6.6, 7.1)	0.6 (0.5, 0.6)	0.4	4.7	96.3
Asthma	5.8 (5.6, 6.0)	3.9 (3.7, 4.1)	1.9	42.0	97.1
Any other heart condition	5.1 (4.9, 5.3)	2.1 (1.9, 2.2)	1.2	24.5	97.2
Anemia	4.6 (4.4, 4.8)	3.0 (2.8, 3.2)	1.9	30.0	97.2
Stomach, duodenal, or peptic ulcer	3.3 (3.1, 3.5)	0.6 (0.5, 0.6)	0.3	14.3	98.3
Angina (chest pain)	2.6 (2.4, 2.8)	0.3 (0.2, 0.3)	0.2	7.8	98.7
Neuropathy	2.2 (2.0, 2.3)	4.5 (4.3, 4.7)	4.0	15.9	97.1
Gallstones	1.7 (1.6, 1.8)	0.7 (0.7, 0.8)	0.2	43.9	99.3
Posttraumatic stress disorder	1.5 (1.4, 1.7)	1.3 (1.2, 1.4)	1.0	20.7	98.8
Any other hepatitis	1.3 (1.2, 1.5)	0.5 (0.4, 0.6)	0.4	8.2	99.2
Hepatitis B	1.1 (1.0, 1.3)	0.1 (0.1, 0.2)	0.1	8.8	99.4
Seizures	0.9 (0.8, 1.0)	0.4 (0.4, 0.5)	0.2	29.3	99.5
Heart attack	0.7 (0.6, 0.8)	0.1 (0.1, 0.2)	0.1	19.3	99.7
Pancreatitis	0.7 (0.6, 0.8)	0.2 (0.1, 0.2)	0.1	25.5	99.7
Stroke	0.5 (0.5, 0.6)	0.2 (0.2, 0.3)	0.2	9.1	99.7

* Sample size varies from 37,328 to 37,696 due to missing values.

**Figure 2:**
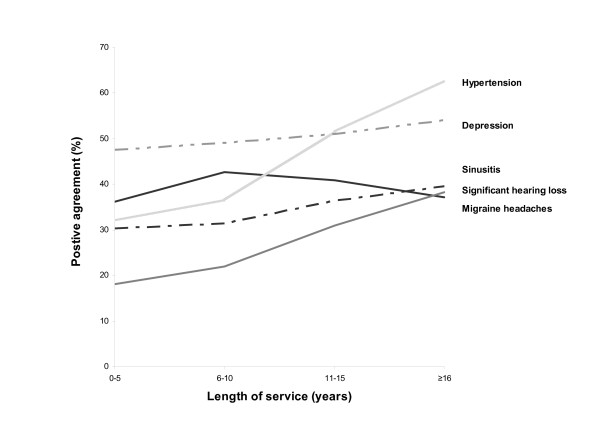
Positive agreement by length of service for the five most-prevalent medical conditions.

Overall, prevalence and agreement values varied with increasing length of service (Table 3). In most cases, both prevalence and positive agreement increased with longer time in service (Table 3, Figure 2). For example, the prevalence of hypertension based on self-report increased from 4.2 percent among those with 0–5 years of service, to 17.2 percent among those with ≥ 16 years of service. Figure 2 shows the five most prevalent conditions over length of service, positive agreement for hypertension increased considerably with greater length of service, from 32 percent to 63 percent (Figure 2).

**Table 3:** Prevalence by length of service of most commonly reported medical conditions via self-report, electronic medical record, either self-report or electronic medical record, and both self-report and electronic medical record among active-duty Millennium Cohort participants

Length of Service (years)																
	0–5 *n *= 7,183^†^				6–10 *n *= 8,516^†^				11–15 *n *= 10,179^†^				≥ 16 *n *= 10,574^†^			
				
Medical condition*	% self	% elec	% either	% both	% self	% elec	% either	% both	% self	% elec	% either	% both	% self	% elec	% either	% both
Sinusitis	8.7	12.8	18.3	3.3	11.7	12.9	20.8	3.9	15.1	15.0	24.6	5.5	20.6	14.2	28.0	6.9
Migraine headaches	10.8	4.3	12.4	2.7	11.0	5.0	12.5	3.4	10.2	4.1	11.3	2.9	11.3	4.1	12.5	2.9
Hypertension	4.2	3.4	6.4	1.2	7.2	5.0	10.0	2.2	9.6	7.5	12.7	4.4	17.2	13.6	21.2	9.7
Significant hearing loss	4.0	4.4	7.7	0.8	6.0	4.0	8.9	1.1	8.7	5.4	11.9	2.2	15.9	8.8	19.9	4.7
Depression	9.0	9.1	13.8	4.3	6.9	7.3	10.7	3.5	5.6	5.9	8.6	2.9	7.1	5.4	9.2	3.4
Bladder infection	8.5	0.9	9.1	0.3	6.4	0.8	7.0	0.2	6.1	0.4	6.4	0.1	6.8	0.3	7.0	0.1
Asthma	5.7	4.3	7.8	2.2	5.3	4.0	7.3	2.0	5.4	3.6	7.1	1.9	6.6	3.9	8.3	2.1
Any other heart condition	3.2	1.0	3.8	0.3	4.1	1.6	5.0	0.6	4.9	2.0	6.1	0.8	7.3	3.2	9.0	1.4
Anemia	5.8	3.4	7.8	1.4	4.8	2.4	6.2	1.0	4.3	3.1	6.2	1.2	4.0	3.0	6.0	1.1
Stomach, duodenal, peptic ulcer	2.4	0.3	2.6	0.2	2.7	0.4	2.9	0.2	3.1	0.6	3.3	0.3	4.6	0.8	5.0	0.4
Chronic bronchitis	3.4	4.7	7.6	0.5	3.1	4.0	6.6	0.6	2.8	3.7	6.1	0.4	3.7	3.8	7.0	0.5

* Most prevalent conditions among all Cohort members; 3 percent or greater self-reported prevalence.

^† ^Sample size varies slightly for each condition due to missing values.

## Discussion

Health survey research obtaining outcome and risk factor information relies heavily on the ability of participants to correctly and specifically self-report their medical histories. Previous studies that have looked at the reliability of these data have focused on one or few conditions simultaneously. The Millennium Cohort questionnaire contains 38 clinician-diagnosed medical conditions self-reported by the participant, which were compared with diagnostic codes from available electronic medical records. The most commonly observed conditions from both data sources were sinusitis, migraine headaches, hypertension, hearing loss, and depression. Prevalence for most conditions was found consistently lower in the electronic medical records than by self-report. Negative agreement between self-report of medical conditions and electronic medical record data was quite high, whereas positive agreement was relatively low, increasing with longer observation periods of objective data.

The choice to use positive and negative agreement rather than other measures, such as the kappa statistic or sensitivity and specificity, was driven by inherent limitations in the applicability of these measures to the current study. The results of this study provide insight into the degree of concordance between self-reported and electronic medical record data in a predominantly healthy, young, working population. Results also illustrate changes in positive and negative agreement with length of time in military service, which, in this study, is equivalent to length of time of accrued medical record data. The observed variability in positive and negative agreement across diagnostic categories highlights the importance of using multiple data sources to assess health outcomes when possible. However, for those cases in which objective electronic data are not available, our assessment of diagnostic codes found exclusively in electronic data sources offers information on what would be missed (magnitude and direction of possible biases) using self-reported data alone.

For the most-prevalent medical conditions, positive agreement increased with length of time in service, illustrating that self-reported diagnoses are likely to be reflected in electronic medical records given enough opportunity for capture in health encounter data. However, time in service is largely associated with age. Thus, increasing diagnoses over time is likely the result of a combination of increasing age and increasing data capture, as well as increasing patient understanding of their medical condition(s). Perhaps chronic conditions are a more appropriate assessment of reliability in reporting. Chronic conditions, if diagnosed early, would persist into adulthood and would be reflected in military healthcare databases and thus be concordant with self-report. A diagnosis in childhood such as sinusitis, however, would not be in the military healthcare databases and would likely be reported on the survey as a diagnosis by the Cohort member thus explaining the reason for lower positive agreement. The lower positive agreement with acute conditions such as asthma may also be a result of diagnosis prior to military service. Alternatively, conditions such as kidney failure requiring dialysis, cirrhosis, and emphysema should be obvious to the person and happen later in life where surveillance with medical healthcare data would indicate such a disorder. These data suggest differing methods of ascertainment dependent upon the condition being studied.

There are practical reasons that may explain some of the lower concordance measures between self-report and electronic medical outcomes. A complete description of the medical condition(s) may not have been sufficiently addressed by the medical practitioner at the time of diagnosis, allowing the patient's perception to differ from what was medically coded. Patients may be in the diagnostic or 'rule-out' phase of an explanation for their ill health and may report conditions for which they have been tested, but not diagnosed, or may self-diagnose in the absence of a satisfactory explanation for their health complaint. Inadequate patient-clinician communication may also account for some of the disagreement noted in this study. Additionally, the "health literacy" of patients may also explain reduced recognition of listed medical outcomes in the survey if the knowledge proficiency for some medical conditions vary [27]. Still, one would argue that with good patient-clinician communication, patients will recognize their diagnosed conditions, and may or may not recognize conditions with which they have not been diagnosed, both leading to an increase in positive and negative agreement. Further, it is not possible to know if an individual self- managed a condition such as migraines without consulting a medical professional. Although the question stated, "Has your doctor or other health professional EVER told...", it is possible that the participant marked affirmative thus impacting agreement between data sources. To a lesser degree, low concordance in other rare conditions may represent inaccurate ICD-9-CM codes within the medical records.

The method for selecting ICD-9-CM codes to represent each condition may have also affected the rates of agreement. The codes presented in Additional file 1 were chosen by two expert reviewers to reflect, in their opinion, the presence of each medical condition, while being neither too broad nor too narrow in definition. If, for example, the selected codes more broadly defined a condition, more cases would be identified in the electronic record, thereby increasing positive agreement but decreasing negative agreement. Conversely, if the codes were narrowly defined, fewer cases would be identified in the electronic record, increasing negative agreement and decreasing positive agreement. To best objectively accomplish this task, the expert clinician researchers chose codes most appropriate for each condition blinded to the diagnostic codes selected by the other. Discrepancies were resolved through discussion.

Another inherent problem with using ICD-9-CM coding for concordance studies is that ICD-9-CM codes are not uniquely related to only one condition. Furthermore, the medical conditions used in this study are not mutually exclusive of each other. For example, myocardial infarctions are a result of coronary heart disease, and therefore, a number of codes used to identify a myocardial infarction (i.e., 410.xx, 411.0, and 412) are also associated with coronary heart disease. If a patient had an ICD-9-CM code of 412 in his or her medical record, and reported having had a heart attack, but not coronary heart disease, the net effect would be an increase in positive agreement for heart attack, but a decrease in this same measure for coronary heart disease. This limitation may help to explain the low concordance for some of the medical conditions analyzed in this study.

Further explanation for disagreement between self-report and objective record data may be due, in part, to the electronic database itself. Electronic medical records contain historical data beginning with October 1988 for active-duty service members. Conditions diagnosed prior to this time or prior to military service may not be identified using these records. To reduce the effect of these limitations, the study population was restricted to active-duty service members, and a lengthy period of observation was used. The probability of many of these medical conditions increases with age and age was found to be associated with length of service (correlation = 91.4%). However, length of service in the military will also affect the likelihood of ascertaining a medical encounter in available military electronic records. Individuals with a shorter service length have less opportunity for medical events to be captured in military health system records than those with longer service time. The mean age and length of service for this study population was 31.9 and 11.0 years respectively, whereas the mean age and length of service for those with at least one reported condition was 33.4 and 12.3 years respectively. To adjust for this factor, prevalence and positive agreement for the most commonly reported conditions were stratified by length of service. We further tried to mitigate the vulnerability to prevalence that the kappa statistic would have resulted in by choosing a measure of positive and negative agreement that is independent of the outcome prevalence. These problems should diminish in general and particularly with future data collection efforts in this population. Furthermore, conditions diagnosed prior to military service or inception of electronic medical records that recur or for which follow-up is ongoing will be captured in available data sources. Validation studies of medical records have found evidence of non-reporting and mis-reporting of diagnoses by physicians [25-27]. When experienced coders are employed, ICD-9-CM codes are assigned using information recorded in the medical record, and therefore, any inaccuracies in the medical record will also be reflected in the coding. In addition, other mis-reporting and non-reporting may occur during the coding process. Other issues will remain that will cause misclassification on the part of the study subject and medical record. Such errors, if random, will serve to diminish associations between outcomes and exposures, thus biasing findings toward the null hypothesis. However, given the large sample size of this cohort, this may not be a major problem with regard to missing significant associations since the high level of statistical power will outweigh the potentially smaller effect due to nondifferential misclassification.

The study population used in this investigation is a subset of Millennium Cohort responders and may not be representative of the military or the Cohort. Analyses were limited to active-duty participants because electronic medical record data are not fully available for Reserve and National Guard members. Further, while on active duty, service members have ready access to essentially free medical care in Defense Department facilities and they seldom seek medical care outside the Defense Department health care system. However, it is possible that for some conditions such as mental health disorders where the person may not want the diagnosis on their military healthcare record, a person may go to an outside provider and incur the cost of treatment.

Despite these limitations, this study has many strengths. Pairing Millennium Cohort data with available electronic medical records allowed examination of a wide range of self-reported medical conditions in a large, working population. Unlike most previous studies of this kind that have focused on older populations, the current study was conducted on a relatively young adult working population. Few epidemiological studies to date have had the resources to investigate the concordance of self-reported and electronic medical record data on such a broad range of conditions in a population of this size. In addition, the electronic medical record data were relatively complete for the available time frame among active-duty personnel. What may be most important about this current report is what these data suggest, in a broad way, that self-reported medical data may be sufficient for ruling out history of a particular condition as suggested by the high negative agreement values. Further, that prevalence studies may be best served by using an objective measure of medical conditions found in electronic healthcare records. Finally, the Cohort itself has been shown to be well-representative of its target population, relatively free of response biases, and to have strong reliability metrics [15,17,18,20-23,28].

## Conclusion

In summary, this article highlights the research challenges of self-reported medical outcomes data and also underscores the potential limitations of electronic medical record data. Data integrity increased with length of observation within the medical record data. This study demonstrated that electronic diagnoses generally agreed with self-reported medical conditions, but more accurately represented the absence of disease over the presence of disease. As in the Millennium Cohort Study, health researchers who rely on self-reported medical conditions should consider using multiple data sources to assess health outcomes when possible, particularly in young or healthy populations.

## Abbreviations

DoD: Department of Defense; ICD-9-CM: International Classification of Diseases, Ninth Revision, Clinical Modification; HCSR: Health Care Service Record; SADR: Standard Ambulatory Data Record; SIDR: Standard Inpatient Data Record.

## Competing interests

The authors declare that they have no competing interests.

## Authors' contributions

BS, LKC, TCS, PJA, EJB, TIH, GDG, MAKR conceived of the study and participated in the design and coordination of the study; BS, LKC, TCS performed the statistical analysis; All authors participated in interpretation of results and writing of the manuscript; All authors read and approved the final manuscript.

## Pre-publication history

The pre-publication history for this paper can be accessed here:



## Supplementary Material

Additional file 1 Self-reported medical conditions and ICD-9-CM codes. Click here for file
