# Supplementary material for: Challenges of self-reported medical conditions and electronic medical records among members of a large military cohort
Source: BMC Med Res Methodol. 2008 Jun 5;8:37. doi: 10.1186/1471-2288-8-37 (PMC2447848; doi:10.1186/1471-2288-8-37)
Supplement: Additional file 1 — Self-reported medical conditions and ICD-9-CM codes. [file 1471-2288-8-37-S1.doc]

APPENDIX 1. Self-Reported Medical Conditions and ICD-9-CM Codes*

|  |  |
| --- | --- |
| Medical condition | ICD-9-CM code* |
|  |  |
| Hypertension (high blood pressure) | 401.0, 401.1, 401.9, 402.xx, 403.xx, 404.xx, 405.xx, 437.2, 642.x, 997.91 |
| Coronary heart disease | 410.xx, 411.xx, 412, 414.0x, 414.1, 414.10, 414.11, 414.12, 414.8, 414.9, 996.03, E942.4 |
| Heart attack | 410.xx, 411.0, 412, 414.8, 429.7x |
| Angina (chest pain) | 413.x, 411.1 |
| Any other heart condition | 391.x, 392.0, 393 to 398.xx, 420.xx to 429.xx |
| Sinusitis | 461.x, 473.x, 993.1 |
| Chronic bronchitis | 490, 491.0, 491.1, 491.2x, 491.8, 491.9 |
| Emphysema | 491.2x, 492.0, 492.8 |
| Asthma | 493.xx, 519.1 (prior to 01 Oct 2003) |
| Kidney failure requiring dialysis | 585 with (39.27, 39.95, 39.42, or 54.98) or 585 with (01844, 36145, 36800, 36810, 36815, 36831, 36832, 36833, 36838, 49420, 49421, 75790, 90935, 90937, 90940, 90945, 90947, 90999, 93990, or 99512)^†^ |
| Bladder infection | 595.0, 601.3, 098.11, 098.31, 112.2, 131.09, 595.2, 595.4, 032.84, 016.1 |
| Pancreatitis | 072.3, 577.0, 577.1 |
| Diabetes or sugar diabetes | 250.x, 271.4, 357.2, 362.0x, 366.41, 648.0, V58.67 |
| Gallstones | 560.31, 574.x |
| Hepatitis B | 070.2, 070.3, V02.61 |
| Hepatitis C | 070.41, 070.44, 070.51, 070.54, 070.7x, V02.62 |
| Any other hepatitis | 070.0, 070.1, 070.43, 070.49, 070.52, 070.53, 070.59, 070.6, 070.9, 072.71, 091.62, 130.5, 570, 571.1, 571.4x, 573.1, 573.2, 573.3 |
| Cirrhosis | 571.2, 571.5, 571.6 |
| Rheumatoid arthritis | 714.0 to 714.3x, 714.81 |
| Lupus | 373.34, 695.4, 710.0 |
| Multiple sclerosis | 340 |
| Crohn’s disease | 555.x |
| Stomach, duodenal, or peptic ulcer | 531.x to 533.x |
| Ulcerative colitis or proctitis | 556.x |
| Significant hearing loss | 388.12, 388.2, 389, 389.0x, 389.1x, 389.2, 389.8, 389.9 |
| Migraine headaches | 346.x, 625.4 |
| Stroke | 430 to 434.x, 436 to 438.xx, 997.02 |
| Neuropathy/reduced sensation hands/feet | 353.x to 357.xx |
| Seizures | 345.x (except 345.6), 780.3, 780.39 |
| Sleep apnea | 780.51, 780.53, 780.57 |
| Anemia | 280.x to 285.xx |
| Thyroid condition other than cancer | 240.x to 246.x |
| Cancer | 140.x to 208.x |
| Chronic fatigue syndrome | 780.71 |
| Depression | 296.2 to 296.7, 298.0, 300.4, 307.42, 307.44, 309.0, 309.1, 309.28, 311, V11.1 |
| Schizophrenia or psychosis | 290.xx to 295.xx, 297.x, 298.x, V11.0 |
| Manic-depressive disorder | 296.0 to 296.8x |
| Posttraumatic stress disorder | 308.x, 309.8x |
|  |  |

^*^ ICD-9-CM, *International Classification of Diseases*, Ninth Revision, Clinical Modification

^†^ CPT®, *Current Procedural Terminology*, procedure codes.
